# Assessing dengue transmission risk and a vector control intervention using entomological and immunological indices in Thailand: study protocol for a cluster-randomized controlled trial

**DOI:** 10.1186/s13063-018-2490-1

**Published:** 2018-02-20

**Authors:** Hans J. Overgaard, Chamsai Pientong, Kesorn Thaewnongiew, Michael J. Bangs, Tipaya Ekalaksananan, Sirinart Aromseree, Thipruethai Phanitchat, Supranee Phanthanawiboon, Benedicte Fustec, Vincent Corbel, Dominique Cerqueira, Neal Alexander

**Affiliations:** 1 Norwegian University of Life Sciences, Ås, Norway; 2 Khon Kaen University, Khon Kaen, Thailand; 3 HPV & EBV and Carcinogenesis Research Group, Khon Kaen University, Khon Kaen, Thailand; 4 Office of Disease Prevention and Control, Region 7, Khon Kaen, Thailand; 5 PT Freeport Indonesia/International SOS Indonesia, Kuala Kencana, Indonesia; 6 Kasetsart University, Bangkok, Thailand; 7 Université de Montpellier, Montpellier, France; 8 Institut de Recherche pour le Développement (IRD), Maladies Infectieuses et Vecteurs, Ecologie, Génétique, Evolution et Contrôle (MIVEGEC, UM1-CNRS 5290-IRD 224), Montpellier, France; 9 MRC Tropical Epidemiology Group, London, UK

**Keywords:** Dengue monitoring, Entomology, Immunology, Dengue index, Risk assessment, Vector control

## Abstract

**Background:**

Dengue fever is the most common and widespread mosquito-borne arboviral disease in the world. There is a compelling need for cost-effective approaches and practical tools that can reliably measure real-time dengue transmission dynamics that enable more accurate and useful predictions of incidence and outbreaks. Sensitive surveillance tools do not exist today, and only a small handful of new control strategies are available. Vector control remains at the forefront for combating dengue transmission. However, the effectiveness of many current vector control interventions is fraught with inherent weaknesses. No single vector control method is effective enough to control both vector populations and disease transmission. Evaluations of novel larval and adult control interventions are needed.

**Methods/design:**

A cluster-randomized controlled trial will be carried out between 2017 and 2019 in urban community clusters in Khon Kaen and Roi Et cities, northeastern Thailand. The effectiveness of a pyriproxyfen/spinosad combination treatment of permanent water storage containers will be evaluated on epidemiological and entomological outcomes, including dengue incidence, number of female adult dengue vectors infected or not infected with dengue virus (DENV), human exposure to *Aedes* mosquito bites, and several other indices. These indices will also be used to develop predictive models for dengue transmission and impending outbreaks. Epidemiological and entomological data will be collected continuously for 2 years, with the intervention implemented after 1 year.

**Discussion:**

The aims of the trial are to simultaneously evaluate the efficacy of an innovative dengue vector control intervention and developing predictive dengue models. Assessment of human exposure to mosquito bites by detecting antibodies generated against *Aedes* saliva proteins in human blood samples has, so far, not been applied in dengue epidemiological risk assessment and disease surveillance methodologies. Likewise, DENV detection in mosquitoes (adult and immature stages) has not been used in any practical way for routine disease surveillance strategies. The integration of multiple outcome measures will assist health authorities to better predict outbreaks for planning and applying focal and timely interventions. The trial outcomes will not only be important for Thailand, but also for the entire Southeast Asian region and further afield.

**Trial registration:**

ISRCTN, ISRCTN73606171. Registered on 23 June 2017.

**Electronic supplementary material:**

The online version of this article (10.1186/s13063-018-2490-1) contains supplementary material, which is available to authorized users.

## Background

Dengue fever is the most common and widespread arboviral disease in the world, with an estimated four billion people in at least 128 countries at risk of infection [1]. The exact global burden of dengue is not known, but there are estimates of about 390 million infections annually, of which only a minority (~ 25%) manifest clinically [2]. Yearly mortality figures of > 20,000 deaths have been reported [3]. Dengue fever and other arboviral diseases, such as Zika and chikungunya, are transmitted to humans primarily by *Aedes aegypti* and *Aedes albopictus* mosquitoes. There is currently no specific treatment for dengue and only recently has a vaccine been licensed, but it does not confer full protection for all virus serotypes [4]. Even with effective therapies and vaccines, vector control will likely remain important to curtail disease incidence and outbreaks. Perennial dengue incidence varies seasonally, and dengue outbreaks occur periodically in most endemic countries [5]. Infection of one of the four dengue virus serotypes (DENV1—4) typically confer lifelong protective homotypic (type-specific) immunity as well as production of more time-limited cross-reactive heterotypic neutralizing antibodies [6]; however, antibody-dependent enhancement may result in a second DENV serotype infection inducing a more severe clinical course [5].

For improved dengue control, reliable epidemic forecasting systems for early detection of temporal anomalies in disease incidence are needed, as well as more effective control strategies that affect both entomological and epidemiological endpoints. Sensitive surveillance tools do not exist today, and only a small handful of new control strategies are available [7–14]. For example, although temephos — the most commonly used chemical-based vector control method, used against immature mosquito stages — may be effective in reducing entomological indices, there is no evidence showing it reduces dengue transmission [7].

There is a compelling need to develop cost-effective approaches and practical tools that can reliably measure real-time dengue transmission dynamics that enable more accurate and useful predictions of outbreaks. Currently, there is no universally accepted definition of what constitutes an outbreak [15]. This complicates the interpretation of early detection of cases that exceed expected normal seasonal variations. In many endemic countries, a dengue outbreak is declared when the number of reported cases during a specific time period (week or month) surpasses the historical average of the preceding 5 to 7 years above two standard deviations (SD), known as the *endemic channel* [5]. Outbreak definitions vary depending on how the historical average is calculated, which may involve, for example, the number of years used, type of mean (e.g., monthly or moving mean), whether or not outbreak years are included, how the critical threshold is calculated (e.g., ±2 SD), and criteria used to define the outbreak (e.g., time above the threshold before a response is triggered) [15]. Ideally, when an outbreak alert has been triggered, standard vector control strategies should be implemented. However, current early warning systems and detection of outbreaks are usually neither accurate nor timely enough to initiate effective control interventions (outbreak response) to curb increased transmission after it has begun [11].

Various entomological indices are used to measure dengue vector infestation in and around structures (homes, buildings, etc.). However, these indices are seldom sensitive enough to precisely estimate dengue transmission risk or predict impending outbreaks [16, 17]. The Stegomyia indices, i.e., House index (HI, proportion of *Aedes* positive houses) and Container index (CI, proportion of *Aedes* positive containers) were developed nearly a century ago [18], followed by the Breteau index (BI, number of *Aedes* positive containers per 100 houses) [19]. These three measures are currently the most commonly used indices to assess dengue vector larval habitat infestations. They are relatively easy to measure, but are generally not correlated with disease incidence or outbreak risk [16]. In the 1990s, Focks et al. explored the use of pupal surveys as a potentially more epidemiologically relevant index, correlating total pupal densities with resultant adult densities [20]. This led to further development of entomological thresholds using a pupal/demographic method, ambient temperature, and seroprevalence of dengue antibodies in the population [21]. As a result, container-specific, targeted source reduction was proposed by identifying the relative importance of major types of container habitats with high pupal productivity that contribute significantly to the transmission threshold [21]. However, it remains unclear if targeting only containers that are responsible for the vast majority, say 80–90%, of the pupal production [22–26] is sufficient to have an epidemiological impact on transmission. Other aquatic habitats may be important, such as unusual and cryptic sites, which are typically overlooked during vector control interventions [27, 28]. Furthermore, in many settings, such as in northeastern Thailand and southern Laos, as many as eight to ten of the most productive container types might only produce < 70% of all pupae [29]. Although there are perceived benefits to targeting only the most productive containers, such as reduced time and effort, they may not compensate for ignoring control of other less obvious breeding habitats. The difficulty in finding and effectively treating such cryptic sites can be addressed by using pyriproxyfen, a potent insect growth regulator, which can be transferred between habitats by female *Aedes* mosquitoes during oviposition, a strategy called auto-dissemination [30–32].

A recent study from Iquitos, Peru investigated the relationship between several indicators of *Ae. aegypti* abundance and DENV infection in humans using more than 8000 paired serological samples with corresponding entomological data [17]. The researchers found that indicators based on cross-sectional entomological surveillance, i.e., data from a single survey observation, are of little practical use. On the other hand, longitudinal-based, household-level entomological indicators using data from up to three yearly visits before a 6-month seroconversion period showed that the presence of adult female *Ae. aegypti* in a household increased the risk of DENV seroconversion by approximately 29% compared to households without mosquito vectors. The authors therefore challenged the assumption that most common *Ae. aegypti* indicators provide adequate proxies for DENV risk and transmission [17].

The general response by national dengue control programs to indications of increased disease transmission and possible outbreaks mainly consists of reactive vector control. Typically, control activities involve application of temephos (an organophosphate compound) to domestic water storage containers for larval control and/or peridomestic space spraying with an insecticide, most commonly a pyrethroid-based formulation, for adult control. Although, these interventions may reduce vector populations dramatically, there is no evidence that they reduce dengue transmission substantially [7, 9]. Other possible options for vector control are community-based source reduction campaigns, application of bacteria-based larvicides, larvivorous fish, or copepods, or combinations of these approaches [8, 33–35]. Newer paradigms for *Aedes* vector control include microbial control of human pathogens in adult vectors, such as *Wolbachia* bacteria that shorten the lifespan of mosquitoes [36] and release of transgenic *Ae. aegypti* engineered to carry a dominant lethal gene that suppresses mosquito populations [37]. These novel approaches are currently not recommended for full-scale programmatic deployment by the World Health Organization (WHO) Vector Control Advisory Group, but rather implemented as carefully planned pilot interventions under operational conditions [38]. The effectiveness of many vector control interventions is fraught with inherent weaknesses, e.g., widespread insecticide resistance, quality of delivery, and other operational issues, such as availability and cost of insecticide, dedicated and trained personnel, and appropriate application equipment [39, 40].

The WHO Global Strategic Framework for integrated vector management (IVM) was released in 2004 and recommends a range of interventions, in combination, to increase impact [41]. This means there is no single vector control method that is effective enough to control both vector populations and disease transmission. Combinations of larval control interventions, such as mixtures of pyriproxyfen (an insect growth regulator) and spinosad (a biopesticide) have been evaluated. This combination reduced larval and pupal relative densities by 90% for at least 4 months in the French West Indies [42]. Pyriproxyfen, even in minute doses, can induce complete inhibition of adult emergence for several weeks after treatment [43]. Pyriproxyfen used alone and applied to storm drains in Colombia reduced dengue cases by 80% [44]. The benefit of using these two compounds in combination is that they have different modes of action and that pyriproxyfen targets the pupal stage while spinosad is active against larval stages. Both compounds have very low toxicity for humans and most other non-target fauna [45, 46]. The WHO draft on global vector control response for 2017–2030 [47] builds on the IVM approach but places stronger emphasis on enhancing human capacity and health education, increasing research and innovation by strengthening infrastructure, and increasing intersectoral and interdisciplinary action. The targets of the global response are to reduce mortality and incidence due to all vector-borne diseases globally relative to 2016 by at least 75% and 60%, respectively.

In view of the preceding discussion, this study aims to assess a specific vector control intervention and to contribute to the development of a practical early warning system that can more accurately predict changes in dengue transmission and impending outbreaks. The trial will determine the efficacy of a pyriproxyfen/spinosad combination in water storage containers to reduce entomological risk indicators and dengue incidence. The hypothesis is that the study arm receiving the combination treatment in household water storage containers will have a lower density of adult female *Ae. aegypti* per house, both indoors and outdoors, compared to the study arm receiving an alternative intervention involving normal governmental action. Furthermore, the study aims to determine one or more entomological indices and an immunological index that best predicts dengue incidence for the study area.

## Methods/design

### Objectives

The specific objectives of this trial are to:

1. Assess the effect of periodically treating water storage containers with a pyriproxyfen/spinosad combination on entomological and epidemiological outcomes
2. Determine the most accurate and precise index or indices to predict variation in dengue incidence in time

### Trial design

A stratified, cluster-randomized controlled trial is designed to study the effect of a vector control intervention in households located in pre-selected clusters in two urban areas in northeastern Thailand. Each cluster is randomized to one of two arms: intervention clusters receiving treatment of water containers with a pyriproxyfen/spinosad combination, and control clusters not receiving any intervention from this project. Control areas will rely on normal operational vector control interventions performed by the local public services. Randomization of arms is stratified by city (Khon Kaen and Roi Et, i.e., two levels). Stratification is done because there are potential differences between the two cities that may affect the outcomes (e.g., population size, and regional importance in terms of travel, commerce, services, health care, and education); therefore, stratification may reduce the residual statistical error when one compares the two arms. Including two cities may also alleviate problems of low incidence caused by the spatial and temporal variation in dengue transmission; i.e., one area functions as a backup if there are few cases in the other. Including two cities should also increase the generalizability of the trial.

A cluster design is considered the best option because the intervention is not performed on the individual level, but is rather a spatial, area-wide approach involving treatment of containers in and immediately around each house and property in a study area (cluster). Within a household, it is not feasible to randomize some individuals to one intervention and other individuals to another. Furthermore, the entomological outcomes, both primary (Adult index) and secondary (e.g., Pupal index per person, and the Breteau index), will be estimated on a household level. We are using the larger clusters rather than single houses because (1) there may be mass (area) effects of the interventions, whereby entomological indices in each house may depend partly on the abundance of mosquitoes in neighboring ones, and (2) entire clusters having the same intervention more closely resembles how the intervention would be implemented, should it be scaled up.

### Setting

**Fig. 1:**
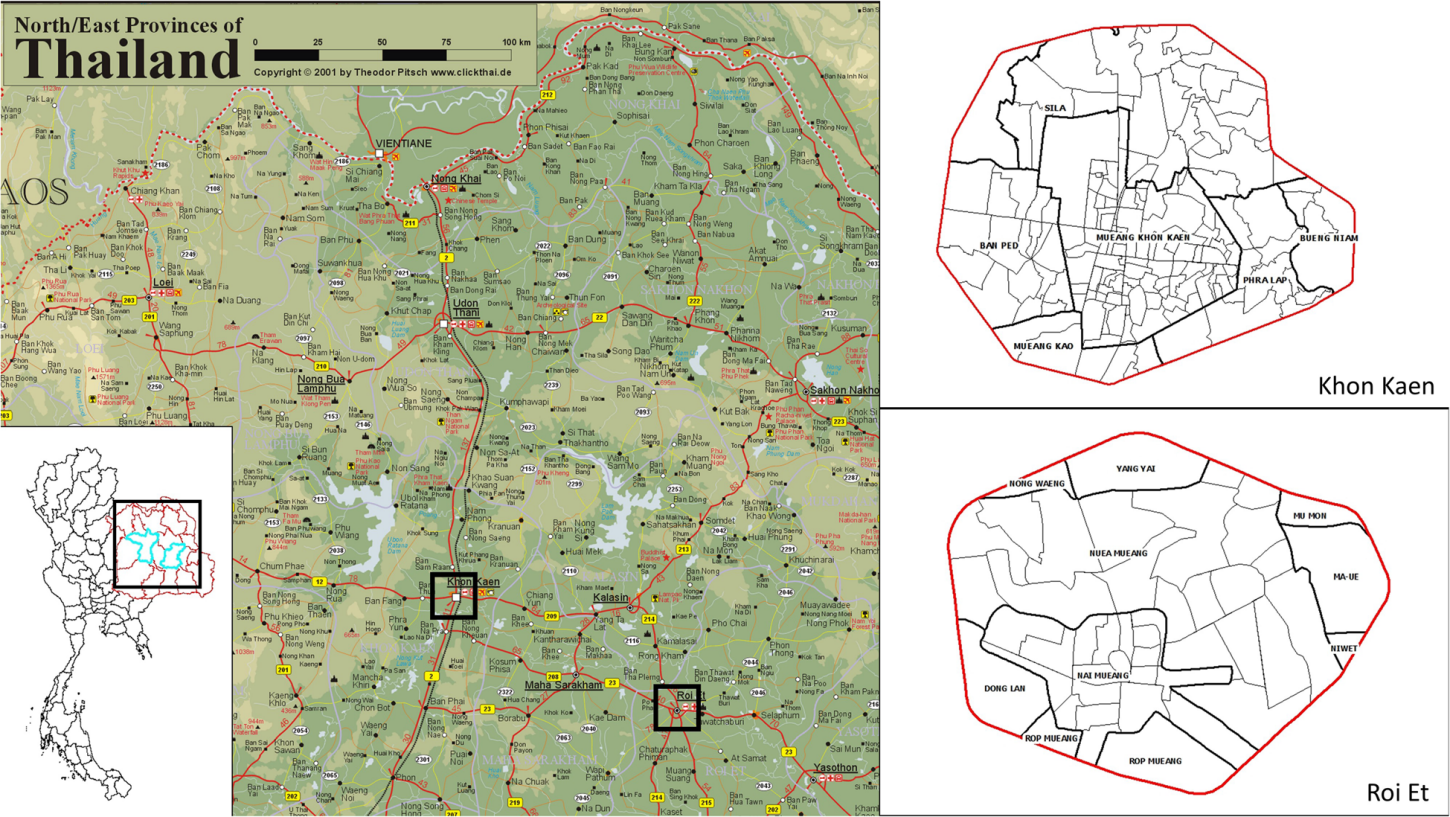
Study locations in northeastern Thailand. The red borders around the cities (right insets) are the respective ring roads.

The trial is carried out in two urban areas, Khon Kaen (N16.440236, E102.828272) and Roi Et (N16.055637, E103.652417) cities in northeastern Thailand (Fig. 1). Khon Kaen is the capital city of the province with the same name. The province has an area of ~ 10,900 km^2^, divided into 26 districts and a population of 1,741,980 in the 2010 national census [48]. Khon Kaen district is the largest by area and population, with a population of around 400,000 over an area of 953.4 km^2^ (population density 416 persons/km^2^). The district is divided into 17 sub-districts with 272 villages. In 2016, there was a population of 269,247 in six sub-districts that make up greater Khon Kaen (within the ring road) with a resident density of 2500/km^2^ in the central parts. Roi Et is the capital city of Roi Et province and is divided into 20 districts covering a total area of 8300 km^2^ with a population of 1,084,985 in 2010 [48].The largest district is also called Roi Et, and it has ~ 160,000 inhabitants, covers an area of 493.6 km^2^, and has a population density of approximately 311 inhabitants/km^2^. There are 15 sub-districts and 195 villages in Roi Et district. The largest sub-district is Nai Mueang Roi Et municipality with a population of approximately 34,000 inhabitants. To delimit the study area, only villages completely within each city’s primary access ring road are selected. We use the English word “village”, although most are urban divisions. In Khon Kaen, there are 162 villages in six sub-districts within the ring road. In Roi Et, there are 56 villages in nine sub-districts within the ring road, although only 39 have clearly demarcated administrative boundaries.

Between 2006 and 2016, the total number of reported dengue cases (uncomplicated and severe categories) reported in Khon Kaen province was 15,195 (mean 1381 cases/year, range 439–3014), providing an incidence rate of 76.7 cases/100,000 population. In Khon Kaen district, the number of cases during the same period was 7209 (mean 655 cases/year, range 204–1705), with an estimated incidence of 455.3 cases/100,000. The corresponding numbers for Roi Et province for the same period were 20,174 total cases (mean 1834 cases/year, range 402–4141) and an incidence of 140.2 cases/100,000. In Roi Et district, 3956 cases were reported during the period (mean 360 cases/year, range 71–914) with an incidence of 329.1 cases/100,000. All dengue case data were provided by the Office of Disease Prevention and Control Region 7, Khon Kaen, Ministry of Public Health.

### Outcomes

The primary outcome is the Adult index (AI, the number of female adult *Ae. aegypti* and *Ae. albopictus* collected per house) (Table 1). The AI is based on combined indoor and outdoor collections using mechanical, battery-powered aspirators for 30 min (2 × 15 min) by staff of the local public health departments. The AI for each species will be recorded separately, although the general expectation is to find a predominance of *Ae. aegypti* in all urbanized clusters.

**Table 1:** Primary and secondary outcome measures and other entomological indices

Outcome	No.	Index abbreviation	Index name	Description	Unit	Frequency of data collection	Details
Primary outcome	1	AI	Adult index	Number of female adult *Ae. aegypti* and *Ae. albopictus* per house collected both indoors and outdoors	No./house	Once every 4 months in all households (HHs) and once every month in 3 HHs per cluster (the same ones each time)	Adult collections using a mechanical battery-powered aspirator for 30 min per house (indoors and outdoors)
Secondary outcomes	2	DIR	Dengue incidence rate	Number of confirmed dengue cases/observation days of household populations	Rate	Weekly	VHVs detect fever cases. Hospital and project staff collect blood samples
	3	MEI	Mosquito exposure index	(1) Differential optical density for antibodies to *Ae. aegypti* saliva (2) Proportion above the immune threshold for this assay	(1) Number (2) %	Once every 4 months in all HHs and once every month in 3 HHs per cluster (the same HH as for the AI)	Recurring blood spots from two persons per HH taken on filter paper. IgG antibody response (positive or negative) to the *Ae. aegypti* Nterm-34 kDa salivary peptide
	4	IAI	Infected adult index	Number of DENV-infected adult female *Ae. aegypti* and *Ae. albopictus*	No./house	Once every 4 months in all HHs and once every month in 3 HHs per cluster	Based on adult mosquito collections indoors using a mechanical battery-powered aspirator for 15 min per house and DENV detection in individual mosquitoes
	5	ASTI	Adult sticky trap index	Total number of *Ae. aegypti* and *Ae. albopictus* females collected by sticky traps per month	No./trap/month	7 consecutive days per month	Adult mosquitoes collected by sticky traps baited with hay infusion for 7 days every month
	6	PPI	Pupae per person index	Number of *Aedes* pupae per person	No./person	Once every 4 months in all HHs and once every month in 3 HHs per cluster	From immature collections. All pupae collected divided by the number of household participants
	7	BI	Breteau index	Number of *Aedes* positive containers per 100 houses	No./100 houses	Once every 4 months in all HHs and once every month in 3 HHs per cluster	From immature collections. Cluster-level result
Stegomyia indices	8	HI	House index	Proportion of houses positive for immature *Aedes*	%	Once every 4 months in all HHs and once every month in 3 HHs per cluster	From immature collections. Cluster-level result
	9	CI	Container index	Proportion of containers positive for immature *Aedes*	%	Once every 4 months in all HHs and once every month in 3 HHs per cluster	From immature collections. Cluster-level result
Pupal indices	10	IPPI	Infected pupae per person index	Number of DENV infected *Aedes* pupae per person	No./person	Once every 4 months in all HHs and once every month in 3 HHs per cluster	Based on PPI and DENV detection. All infected pupae collected divided by the number of household participants
	11	PHI	Pupae per house index	Number of pupae per house	No./house	Once every 4 months in all HHs and once every month in 3 HHs per cluster	Pupal collections
	12	IPHI	Infected pupae per house index	Number of DENV-infected *Aedes* pupae per house	No./house	Once every 4 months in all HHs and once every month in 3 HHs per cluster	Pupal collections and DENV detection
Adult indices	13	AII	Adult indoor index	Number of *Ae. aegypti* and *Ae. albopictus* females per house indoors	No./house	Once every 4 months in all HHs and once every month in 3 HHs per cluster	Adult collections indoors using a mechanical battery-powered aspirator for 15 min per house
	14	IAII	Infected adult indoor index	Number of DENV-infected *Ae. aegypti* and *Ae. albopictus* females per house indoors	No./house	Once every 4 months in all HHs and once every month in 3 HHs per cluster	Based on AII and DENV detection
	15	AOI	Adult outdoor index	Number of *Ae. aegypti* and *Ae. albopictus* females per house outdoors	No./house	Once every 4 months in all HHs and once every month in 3 HHs per cluster	Adult collections outdoors using a mechanical battery-powered aspirator for 15 min per house
	16	IAOI	Infected adult outdoor index	Number of DENV-infected *Ae. aegypti* and *Ae. albopictus* females per house outdoors	No./house	Once every 4 months in all HHs and once every month in 3 HHs per cluster	Based on AOI and DENV detection
	17	IASTI	Infected adult sticky trap index	Number of DENV-infected *Ae. aegypti* and *Ae. albopictus* females per sticky trap	No./trap/week	Once every 4 months in all HHs and once every month in 3 HHs per cluster	Based on ASTI and DENV detection
Premise index	18	PCI	Premise condition index	Degree of shade + condition of house + condition of yard	Number (min = 3, max 9)	Once every 4 months in all HHs	Observation criteria [90]

*VHV* village health volunteer, *IgG* immunoglobulin G

The secondary outcomes are the dengue incidence rate (DIR), mosquito exposure index (MEI), infected adult index (IAI), adult sticky trap index (ASTI), pupae per person index (PPI) and BI (Table 1). Dengue cases (incidence) is a secondary endpoint, because the sample size required to detect a difference would be unfeasibly large.

In addition to the intervention-related outcomes, the study will attempt to predict dengue incidence over time using entomological and immunological indices. The main outcomes for this part are identification of measures (indices) with sufficient accuracy and precision in terms of predicting dengue outbreaks as defined by the Ministry of Public Health.

### Sample size

The sample size is calculated based on data on adult female *Aedes* mosquitoes collected using mechanical aspirators (15 min outdoors and indoors each) from a case-control study in nearby districts during 2016 and 2017. The mean capture was 0.78 mosquito per house (indoors + outdoors). At minimum, 34 clusters are needed to detect a 90% difference in adult female mosquitoes per house with 90% statistical power and a two-sided significance level (α) of 0.05 assuming 10 households are visited three times each after the intervention begins and a between-cluster coefficient of variation of 0.33. Sample size methods for cluster-randomized trials were used [49], as implemented in the ‘clustersampsi’ add-on to Stata® statistical software [50]. The existing data were over-dispersed relative to a Poisson distribution; therefore, to represent a negative binomial distribution with a dispersion parameter (α, or 1/*k*) of 2.03 estimated from the same data, the ‘means’ option was used, with the variance in each arm equal to the mean plus the square of the mean times α [51]. The clusters are split equally between the strata (Khon Kaen and Roi Et). To provide an equal number of clusters in each arm, one extra cluster is added per stratum, i.e., 18 clusters per stratum (city) and 9 clusters per arm in each stratum.

### Eligibility criteria

Eligibility for participation in the trial is determined on four levels: (1) location or village, (2) cluster of houses within village, (3) households within cluster, and (4) individuals within households (household residents) (Table 2).

**Table 2:** Eligibility criteria by location, cluster, household, and individual

Level	Inclusion criteria	Exclusion criteria
Village	- Within ring roads of each city (stratum)	- Area < 0.125 km^2^
	- Populated residential areas	- Number of houses < 100
		- Population < 300
		- Coverage of residential area 70–80% (scattered housing)
		- Non-residential areas, e.g., agricultural fields, airports, industrial areas, commercial areas, (e.g., shopping malls), government offices, lakes, army camps, hospitals, and schools
Cluster	- All points of the cluster are at least 100 m from the nearest point of the village border	
Household	- Households that are permanently inhabited	- Apartment buildings
		- Abandoned houses
	- Households that are built or re-populated during the study period	- Non-permanent households
Individual	- Individuals in households where household head has signed informed consent for household to participate in project	- A travel history outside the village during the previous 7 days
	- Self-reported fever within the last 7 days	- Chronic disease, such as HIV/AIDS, or other health condition that preclude participation in the study
	- Age ≥ 1 year old	- Apparent inability to give informed consent, e.g., due to mental disability

There is a distinction between being included in the final evaluation of endpoints and inclusion for receiving interventions. For example, abandoned houses and non-permanent household structures are not included in evaluation of endpoints, but they may be treated with an intervention if they are located within a radius of 100 m and as feasibly possible

### Recruitment

The 162 villages in Khon Kaen and 39 villages in Roi Et located within the respective ring roads are the sampling frames for each stratum. In each stratum, villages are randomly sampled based on probability proportional to population size, i.e., the population of occupied houses (the target denominator of the primary endpoint). These selected villages will be randomly allocated between the arms (see the section on Assignment of interventions below).

Villages are normally much larger than the target cluster size (10 houses); therefore, to select a starting point for the house selection, a 50 × 50 m grid and a 100-m buffer zone inside of the village perimeter will be superimposed over each village map. The buffer zone of approximately 100 m on the inside of each village border is applied to reduce potential “contamination” (in-flying mosquitoes) from neighboring villages. A random grid cell is selected in each village and 10 houses nearest the centroid of that cell selected. This procedure is followed in each village in this manner as far as practically possible. For example, if a selected household does not want to participate in the study, a neighboring house will be selected.

Informational meetings are held at the sub-district and village administrative levels to provide information about the project and benefits to the communities. Householders are visited and carefully informed about the study, and informed consent is obtained from the household head. A complete enumeration of all participants in the selected clusters will be completed with assistance from the local administration and village health volunteers (VHVs). This enumeration will be done three times during the study, allowing monitoring of potential participants’ discontinuation in the trial. Data on discontinuation, whether due to movement outside the study area or withdrawal of consent, are relevant for the secondary outcomes of DIR and MEI. Reasons for potential discontinuation will be monitored and taken into account in communication strategies to promote retention. In addition, a minor monetary compensation for those who provide blood samples for the mosquito exposure study will promote participant retention to complete follow-up of individuals.

### Interventions

**Fig. 2:**
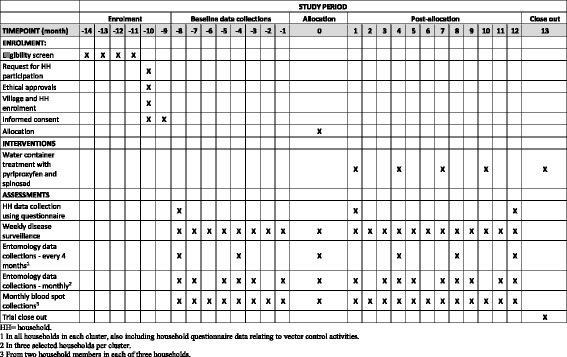
Time schedule of enrollment, interventions, and pre- and post-allocation data collections (based on SPIRIT 2013 figure [91])

Following approximately 10–12 months of baseline data collections, household interventions will begin in the selected intervention clusters (Fig. 2). The intervention specifically targets mosquito immature stages by applying a mixture of pyriproxyfen and spinosad to all permanent household containers, whether indoor or outdoor, found to contain water up to a 10-m perimeter from the house. Pyriproxyfen is an insect growth regulator (insect juvenile hormone analog) that is active against pupal stages, resulting in the inhibition of adult development (preventing emergence). It has low mammal toxicity and is recommended by WHO for vector control [52]. Spinosad is a natural insecticide produced by the soil bacterium *Saccharopolyspora spinosa*. It has a neurotoxic mode of action in insects, but with low mammal toxicity, and it is also recommended by WHO [52]. The doses recommended by WHO for *Aedes* immature mosquito control are 0.01 mg/L active ingredient (a.i.) pyriproxyfen (applied as a 0.5% granule formulation) and 0.1–0.5 mg/L a.i. spinosad (also a 0.5% granule formulation) [45, 53]. Both pyriproxyfen and spinosad have also been assessed and approved by WHO for use in drinking water containers [54, 55]. The reasons for selecting this novel intervention are that the combination of pyriproxyfen and spinosad has not yet been tested in a national dengue vector control program; that it should be effective, easy, and practical to use for national control authorities; and that its combined use reduces the risk of resistance development. The interventions will be implemented by project staff from the Ministry of Public Health, thereby ensuring adherence to intervention protocols.

The combination larvicide will be applied simultaneously to containers every 3 months. A buffer zone of approximately 100 m will be established around the selected intervention clusters. All selected households and other households inside this buffer zone will be treated. As far as feasibly possible, abandoned households, non-permanent households, non-occupied properties, and vacant lots inside the buffer zone will be treated in the same manner.

The households in the other half — the control arm clusters — will not receive any specific intervention initiated by the project. However, for ethical reasons, the comparator, i.e., the control arm, will receive normal governmental dengue control activities. Therefore, during the study period, both intervention and control clusters may be subjected to governmental action as part of the existing national dengue control program response. This may consist of space spraying with pyrethroids in and around a household where a dengue (index) case has been reported, including surrounding houses within a radius of 100 m from an index case. Additionally, larval control with temephos applied to household water-holding containers may occur. Larval control activities depend on the availability of staff, insecticides, and time. Although space spraying can be used in clusters of either arm (e.g., if a dengue case is detected), temephos will not be applied in the intervention clusters to avoid biased results and concerns from the public about potential negative effects on water quality. The pyriproxyfen/spinosad combination may be more effective than temephos, particularly since temephos resistance has been detected in *Ae. aegypti* in several sites in Thailand [56].

### Assignment of interventions

#### Sequence generation and implementation

The assignment of intervention (allocation) and control to clusters will be accomplished by two open public lottery events, one in each city (Fig. 2). Allocation will be done several months after and independently from cluster recruitment (Fig. 2). The lottery events will be carried out just before the first intervention. Representatives from each respective sub-district and village, including householders, district village heads, VHVs, and sub-district hospitals, will be invited to attend. Information about dengue and the purpose of the project will be provided. The reasons for randomization, its procedures, and the concepts of intervention and control will be explained. Attendants will also have a chance to ask questions about dengue, vector control, health-seeking behaviors, personal experiences of dengue, and specific details about the project.

Each of the two lotteries will be performed as follows. Small pieces of paper, of the same color and size and thus indistinguishable from one another, numbered from 1 to 18, will be folded and placed in small opaque envelopes and then placed in a bowl. Each number represents a cluster (village). A large screen with the numbered list of village names (from 1 to 18) will be shown above the bowl and visible to all. A person not involved in the study, and accepted by all participants, will be selected to make the draw. Two flip boards with large sheets of paper will be placed on either side of the bowl with the respective headings “Intervention” and “Control” (in Thai). The village on the first paper drawn will be assigned to the intervention arm, the village on the next paper drawn will be assigned to the control arm, and so on. Following the draw, the implications of being in either of the two arms will be discussed and the roles of participants, health volunteers, and sub-district hospital staff will be reviewed. By following this lottery scheme, the interventions are allocated at the same time as the sequence is generated, obviating the need for allocation concealment.

#### Blinding

This study is unblinded for both participants and data collectors because of the nature of the intervention and because it is neither practical nor financially feasible to obtain placebo (blank) granules of pyriproxyfen and spinosad to serve as a control. However, although knowledge of treatment allocation could affect mosquito endpoints (e.g., differential collection efforts), the MEI, which relies on the antibody response to *Ae. aegypti* saliva, should not be affected significantly. In terms of performance bias (i.e., systematic differences in care), dengue incidence is an endpoint, and dengue may initiate contact with health care personnel. However, the subsequent course of the episode does not affect any of the endpoints. In other words, care from health personnel will not affect the dengue diagnosis status, so performance bias should not be a concern.

### Data collection

#### Household questionnaire

Following the consent (see more details later in the paper), the household head will be asked to complete a questionnaire on the normal number of people living in the house, their age and sex, and socioeconomic status, including observations of type and quality of house structure and facilities. The household questionnaire will be repeated *annually*. However, parts of the questionnaire relating to vector control activities will be carried out *every 4 months*.

#### Disease surveillance

VHVs will carry out *weekly* visits at participating households during the 24-month study period. At each visit, household members will be asked about any fever episodes during the preceding week. Body temperature, using an axilla (under-armpit) thermometer, will be measured by the VHVs in all subjects who have reported a recent or current fever. In order to include people who have a fever at times when the VHVs are not visiting, household participants will be asked to call the VHVs by telephone to inform them about this. In that case, the VHV will attempt to visit the house immediately and collect data and temperature from that person. If that is not possible, this person will be included in the next regular VHV visit. Subjects who have or have had a fever (i.e., irrespective of body temperature at the time of the visit) will be brought on the VHV’s motorcycle or by other practical means to the collaborating sub-district hospital and offered a blood test using a commercial rapid diagnostic test kit (RDT: SD BIOLINE Dengue Duo Combo device, cat. no. 11FK46; Standard Diagnostic Inc., Suwon, Korea). This test is designed to detect dengue non-structural protein 1 (NS1) antigen and immunoglobulin M (IgM)/immunoglobulin G (IgG) antibodies. An additional 4-mL blood sample and blood spots will be taken for confirmation of DENV infection and serotype determination. All blood samples will be collected by a certified phlebotomist (or other qualified health staff) in accordance with national guidelines. All blood samples will be transferred to the Department of Microbiology, Khon Kaen University, where they will be processed for serum separation and transferred to a –80 °C freezer to await further processing. RNA extraction will be performed using a QIAamp® Viral RNA Mini Kit (Qiagen, Hilden, Germany) on serum samples. Extracted RNA will be stored at –80 °C for viral detection and sequencing. DENV will be confirmed by nucleic acid detection using reverse transcription polymerase chain reaction (RT-PCR) with DN-F and DN-R primers as described in Shu et al. [57]. Data on potential risk factors, such as patient’s age, travel history, and previous dengue infection history, will be collected at time of blood sampling. Although not part of the outcome factors, tests for Zika [58] and chikungunya [59] infections will be performed using RT-PCR and sequencing for confirmation.

##### Inclusion criteria for individuals

The inclusion criteria for individuals are as follows:

- Self-reported fever within the last 7 days
- Age ≥ 1 year old

##### Exclusion criteria for individuals

The exclusion criteria for individuals are as follows:

- A continuous travel history outside the district during the last 7 days
- Diseases, such as HIV/AIDS or other health conditions, that preclude participation in the study, based on self-evaluation
- Apparent inability to give informed consent, e.g., due to mental disability or other incapacity, or lack of a legally authorized representative

#### Exposure to mosquito bites

To assess the level of exposure to *Aedes* bites, blood spots on filter paper will be taken from each person designated as a fever case (detected during the weekly visits) for immunological analysis. In addition, recurring blood spot collections will be taken from two additional individuals, ideally the same adult and child (5–14 years old) each time. These collections will be done *monthly* in each of three households per cluster and *every 4 months* in all households per cluster. Individuals will be selected based on their availability and willingness to participate over the full course of the study; ideally, they will be individuals who are present at home most of the time. Participants providing blood spots will receive a minor monetary compensation. As the immune background will be variable between individuals, the same individuals are needed to follow changes in their immune response to *Aedes* bites over time. Blood samples will be taken from people in their households by a certified phlebotomist (or other qualified health staff) using a finger prick. Two blood spots (2 × 75 μL) will be placed on filter paper (Protein Card Saver 903™) and stored at 4 °C until further analyses.

Blood samples will be eluted in phosphate-buffered saline (PBS) with 0.1% Tween for 24 h at 4 °C and then stored at –20 °C. The salivary peptide Nterm-34 kDa (Genepep, St Clement de Rivière, France) will be used as an *Aedes*-specific biomarker to quantify the immune response to *Ae. aegypti* mosquito bites by immunoassays [60]. Briefly, the peptide will be coated on a certified plate (MAXISORP®; Nunc, Roskilde, Denmark), and the blood samples will be incubated overnight at 4 °C to allow specific IgG to bind to the salivary peptide. An anti-human IgG secondary antibody enzyme conjugate will be incubated to bind individual IgG attached to the biomarker. Substrate will be added for color development. The level of immune response will be assessed by measuring the absorbance after 120 min at 405 nm (Sunrise™ spectrophotometer, Tecan, Männedorf, Switzerland). Each sample will be compared in duplicate wells and in a blank well (without antigen) to measure non-specific reactions. Individual results will be expressed as a differential optical density (ΔOD) value calculated as ΔOD = OD_x_ − OD_n_, where OD_x_ represents the mean of individual OD values in the two wells containing antigen, and ODn represents the OD value in the well without antigen. Specific anti-Nterm-34 kDa IgG response will be assayed in individuals who have not been exposed to *Ae. aegypti* mosquitoes to quantify the non-specific background antibody level and to calculate the specific immune threshold (TR) as follows:

$$ \mathrm{TR}=\mathrm{mean}\;\left({\Delta \mathrm{OD}}_{\mathrm{unexposed}}\right)+3\mathrm{SD}. $$

The main outcome for this immune response assay will be ΔOD, which is a continuous variable. In addition, a binary outcome will be calculated by considering an individual to be “exposed” if the ΔOD value is higher than the TR calculated from unexposed individuals.

#### Entomological collections

Mosquito collections will be carried out in all participating households *every 4 months* (Fig. 2). In addition, *monthly* collections will be done in the three sentinel households per cluster, using the same households as those used for the blood spot collections for logistical reasons. The following data will be recorded from each household: number of total containers (potential breeding sites, wet or dry), number of containers with water, number of mosquito positive and negative containers (any species), container type, and location (indoors/outdoors) using defined criteria. Mosquito larvae will be collected from all positive containers using a standard larval dipper to determine species composition (both *Ae. aegypti* and *Ae. albopictus* will be identified and recorded). Pupae will be collected using the pupal/demographic survey method [19] and the “five-sweep” net procedure for very large containers [61].

Adult mosquitoes will be collected for 15 min indoors (in living rooms, bedrooms, etc.) and 15 min outdoors (among man-made articles, vegetation, etc.) from each household using a Prokopack mechanical aspirator [62]. Adult mosquitoes will also be collected using stationary sticky lure gravid *Aedes* traps [63] placed in a location where mosquitoes are abundant (based on householders’ knowledge) at four selected households for *7 consecutive days every month*. Specimens will be taken to a laboratory for sorting and identification using a stereomicroscope and morphological keys [64, 65]. Larvae (separated by species) and pupae (separated by species and sex) will be stored in absolute 99.5% ethanol in labeled 1.5-mL Eppendorf® tubes. Blood digestion status (fed or not fed) of female mosquitoes will be determined by external examination of abdomens. Adult mosquitoes (separated by species and sex) will be stored individually in absolute 99.5% ethanol in 1.5-mL labeled Eppendorf tubes. All specimens will be transported to Khon Kaen University and stored at –80 °C until further processing.

#### Virus detection in mosquitoes

Virus detection will be performed on adults and pupae of *Ae. aegypti* and *Ae. albopictus*. The heads and abdomens of adult mosquitoes will be stored separately. Abdomens will be pooled using a pool size of 5–10 individual abdomens depending on abundance. Virus detection will first be performed on all pools; then, if positive, serotype detection will be done on individual mosquitoes (heads). As heads and abdomens cannot be separated in pupae, virus and serotype detection will be done on pools of whole bodies of pupae. The prevalence of infection in the pupal population, based on the proportion of positive pools, will be estimated using previously described methods [66, 67]. The total RNA will be isolated from mosquito specimens using Favorgen® reagent (FavorPrep^TM^ Tissue Total RNA Mini Kit) following manufacturer instructions. The final solution will be stored at –80 °C. DENV presence will be confirmed by real-time quantitative RT-PCR (qRT-PCR) conducted in the LightCycler® 480 Real-Time qPCR System using KAPA SYBR® FAST qPCR Master Mix (2X) Universal [68]. The Master Mix contains an optimized MgCl_2_ concentration. Positive samples will be submitted to a second specific qPCR to determine the DENV serotype [69, 70].

#### Climate data

Climate data, including daily temperature, rainfall, and humidity data, will be collected from permanent weather stations located in Khon Kaen and Roi Et (Department of Meteorology of Thailand). Additionally, four rainfall gauges (three manual and one automatic) and eight temperature-humidity data loggers (iButtons Hygrochron Loggers, DS1923-F5) will be placed in each city at suitable locations to capture local variations.

### Data management

Each participating household will be given a 6-digit identification number (indicating province, village, and household number) and an identification plate (with project name and ID number) attached in a secure location to the house. Each household member will also receive a unique ID number. Data from household questionnaires at household enrollment, entomological collections, blood spot sampling at households and hospitals (venipuncture for dengue positivity confirmation), and disease surveillance data by VHVs will be collected on paper forms. Data will be securely stored in a password-protected central database. All hardcopy and electronic data will be placed in locked spaces or password-protected computers. Data management procedures will be detailed in specific standard operating procedures and can be requested from the corresponding author.

### Analysis

#### Index calculations

For all outcomes, baseline measurements will start in the second half of 2017. Post-intervention measurements will start in the second half of 2018. The following indices or rates will be used:

- *Adult index (AI)*. Number of adult female *Ae. aegypti* and *Ae. albopictus* per house (combined species) collected both indoors and outdoors for 15 min at each location (30 min total collection time), using a battery-driven mechanical aspirator. Collections will occur once every 4 months in all households and once every month in three repeat sentinel households per cluster
- *Dengue incidence rate (DIR)*. Number of confirmed dengue cases divided by observation days of household populations. All household members with a fever will be identified during weekly VHV visits in participating households. Confirmed dengue cases are those febrile patients with a rapid diagnostic test (RDT) positive for NS1, IgM, IgG, or combinations thereof and a subsequent positive laboratory RT-PCR
- *Mosquito exposure index (MEI)*. ΔOD in IgG antibodies to *Ae. aegypti* Nterm-34 kDa salivary peptide using immunoassays, within the sampling scheme described above. Also the proportion for whom this differential optical density is above the TR defined above
- *Infected adult index (IAI)*. Number of DENV-infected adult female *Ae. aegypti* and *Ae. albopictus* per house (combined species) collected both indoors and outdoors for 15 min each, using a battery-driven mechanical aspirator. DENV presence will be confirmed by real-time RT-PCR as described above
- *Adult sticky trap index (ASTI)*. Number of adult female *Ae. aegypti* and *Ae. albopictus* (combined species) collected each month using one sticky trap per house baited with an oviposition attractant hay infusion. Collections will be done in three selected households for 7 consecutive days per month
- *Pupae per person index (PPI)*. Total number of *Aedes* pupae collected in participating households divided by the number of persons in that household. Collections will be done once every 4 months in all households and once every month in three repeat sentinel households per cluster
- *Breteau index (BI)*. Number of immature *Aedes* positive containers per 100 houses measured at the cluster level. Collections will be done once every 4 months in all households and once every month in three repeat sentinel households per cluster

Other indices are described in Table 1.

#### Analysis populations

At the cluster level, analysis will be by intention to treat, i.e., taking the trial arm as that to which each cluster was randomized. At the individual level, people will be taken to have the allocation of the arm in which they are resident at the time of any data contributed. There will be no intention-to-treat analysis, unless, for unforeseen reasons, the Technical Advisory Committee recommends that one be done. A flowchart showing numbers of clusters and average numbers of households per cluster over time will be constructed in accordance with Consolidated Standards of Reporting Trials (CONSORT) guidelines [71]. For the primary analysis, missing data may occur if complete clusters decline to continue in the trial. In this case, the cluster will still be included as long as any data on the primary outcome are available. This does introduce a risk of bias in estimating effectiveness, if loss of clusters is related to performance of the interventions.

#### Statistical methods

For the entomological endpoints, clustering will be taken into account by analyzing summary measures at the level of cluster. The MEI, expressed as a continuous variable, is a characteristic of individual people, not houses, and its main analysis will be by multivariable multilevel modeling with three levels: cluster, individuals within clusters, and measurements (time points) within individuals. As before, the exposure of main interest will be the arm of the trial (intervention versus control). Individual-level covariates will include age (5–14, 15–25, and > 25 years) and sex. Vector control intervention (i.e., arm of the trial) will be used as a covariate at cluster level. Other cluster-level covariates may include abiotic factors (such as rainfall, temperature, and relative humidity) and population density. An additional analysis of MEI will be by summary measures, as for the other endpoints.

For all analyses of summary measures, the arm of the trial will be the exposure of main interest and will be included in regression models as a dichotomous variable. Stratification will be represented by including a dichotomous variable for city. Finally, the baseline value of each outcome, summarized over the pre-intervention rounds, will be included as a categorical variable, with the expectation that this will reduce the residual error.

For each outcome variable, the response variable for the main analysis will be the aggregate value, for each cluster, of the post-baseline measurements. However, for the primary endpoint (AI), an additional analysis will include the values at each post-baseline time point for each cluster, and will include an interaction between the arm of the trial and the time point, with the aim of identifying a possibly waning effect of the interventions.

#### Effect of intervention on primary outcome

For each cluster the total number of adult female *Ae. aegypti* and *Ae. albopictus* and the total number of house visits will be calculated. Taking these as summary measures, a negative binomial regression will be done with the number of mosquitoes as the outcome variable and number of house visits as the exposure (denominator) variable, i.e., with the logarithm of the number of houses as the offset. A logarithmic link function will be used. Hence, the exponential of the coefficient for arm will be the between-arm ratio in AI according to the response variable used.

#### Effect of intervention on secondary outcomes

Dengue incidence in study households will be analyzed using negative binomial regression. The response variable will be the number of dengue cases per cluster, and the exposure will be the person-time at risk. Hence, the analysis will yield rate ratios. Multilevel models will not be used for this outcome, since the number of cases may be too small for them to be fitted robustly. The total number of DENV-infected adult female *Ae. aegypti* and *Ae. albopictus*, i.e., the IAI, will be analyzed in the same way as the AI. The number of adult mosquitoes per sticky trap will be analyzed similarly to the AI, with the exposure variable being the number of traps. This analysis will yield ratios of the ASTI. Pupae per person (number of *Ae. aegypti* pupae/person) will be analyzed similarly to the AI. The denominator of the PPI is the number of persons present per cluster summed over time. For the BI (number of containers with *Ae. aegypti* immatures/100 houses) the denominator for each cluster is the number of house collections during the intervention period. For example, if the same houses are measured at all time points, the denominator is the number of houses times the number of time points.

#### Prediction

The study will attempt to predict dengue incidence over time using entomological and immunological indices based on repeated (monthly) field collections. This will be done in two ways: by predicting the risk of a future outbreak, and by estimating associations between dengue incidence and the indices.

##### Predict the risk of a future outbreak within a week or within a longer lead time

According to the Ministry of Public Health, an outbreak is defined as the number of cases per week exceeding the median number of cases during the last 3–5 years. We will have data on the stated indices 1 week every month, as opposed to every week, so approximately one quarter of the dengue case series data will be able to be used in this analysis.

Using logistic regression, we will develop a prediction rule for the outbreak status (i.e., outbreak or not) in a given week based on data on the indices and on climate, in the previous week or earlier. Climate variables will include rainfall amount and frequency and ambient maximum and minimum temperatures. We will also consider other variables related to housing type and socioeconomic status at the spatial level to be predicted. The aim of the analysis will be to obtain a rule with a high negative predictive value, i.e., with most of the negative predictions being borne out, and with few outbreaks being missed. We will also calculate other operating characteristics such as sensitivity and specificity. We will concentrate on trying to predict outbreaks from one week to the next, i.e., with a lag of 1 week, but will also assess rules for lags up to 4 weeks.

The accuracy of this prediction rule will be assessed by developing it on the majority of the data as a “training” dataset, then evaluating it on the remainder of the data as a “test” dataset. This reduces the tendency to over-estimate the accuracy of prediction when the evaluation is done on the same dataset from which the rule was developed.

##### Estimate associations between dengue incidence and the indices

For this method, we will use Poisson regression and/or time series methods (e.g., autoregressive integrated moving average (ARIMA)) to relate the number of cases per week to our study indices and to climate. Again, the cases in one week will be modeled as a function of data from the previous week or earlier. The associations will be measured in terms of rate ratios or similar coefficients. This analysis will be done using both the incidence in the public health surveillance system and the incidence data from the current study. Associations identified in this analysis may be statistically significant but not of large enough magnitude to enable prediction of outbreak status in the previous section.

### Harms

This study is deemed of minimal risk for the participants. Minimal risk is defined as the probability and magnitude of harm or discomfort anticipated in the research that are not greater in and of themselves than those ordinarily encountered in daily life or during the performance of routine physical or psychological examinations or tests [72]. The vector control interventions, the pyriproxyfen and spinosad formulations, are recommended by WHO for use in disease vector control and in drinking water [46, 53–55]. Hence, adverse events associated with the products are expected to be few. However, an adverse event, should one occur, will be registered by the community-based VHVs through the weekly visits to all households. The project information sheet, given to all participants, also contains contact telephone numbers of the principal investigators and the Khon Kaen University Ethical Committee should any questions or reservations arise. Any adverse event during the trial interventions or trial conduct will be discussed during weekly meetings of the research team at Khon Kaen University. Expedited decisions will be made as to whether any follow-up action is necessary. The opinion of the Technical Advisory Committee (see the following section) will be sought should there be adverse events believed possibly related to the interventions. Based on these considerations, no criteria have been set for discontinuing or modifying the interventions, nor have any trial stopping guidelines been deemed necessary.

### Data monitoring

A Technical Advisory Committee consisting of three independent researchers assumes the role of a Data Monitoring Committee (DMC). The duties of the committee are to stay informed about the progress of the trial; provide advice to the research team when needed; assist in solving ethical issues and unforeseen or adverse events; and determine any potential termination of the trial. This committee is independent and will not benefit from the trial or otherwise influence the trial. The terms of reference of the Technical Advisory Committee can be accessed from the corresponding author. No interim analysis is planned.

### Auditing

There will be no formal auditing of this trial.

### Confidentiality

As described above, the personal information of enrolled participants will be stored in a safe website ensuring confidentiality before, during, and after the trial. Analysis and publication of the results will ensure that no identifiable information is released.

### Ancillary and post-trial care

This trial is deemed of minimal risk to study participants. Therefore, there are no provisions for ancillary or post-trial care or for compensation to those who suffer harms from trial participation, beyond the existing Thai social security system.

### Dissemination policy and access to data

Results from this trial will be published in open access, peer-reviewed journals. The presentation of the final results of this trial will follow the CONSORT 2010 statement and the extension to cluster-randomized trials [71] and, if needed, extensions on non-pharmacological interventions and pragmatic designs [73, 74]. This study protocol followed the recommendations of items to address in a clinical trial protocol (Additional file 1)and the minimum trial registration information of WHO (Additional file 2), in addition to what was registered in the primary ISRCTN registry. Access to the trial dataset will be made available upon publication of results. Access to data will also be archived and made available through the Norwegian University of Life Sciences and the Norwegian Centre for Research Data (http://www.nsd.uib.no/nsd/english/) after the project has officially ended. Results will be communicated to trial participants in easy-to-read local language pamphlets and through post-project dissemination events. Access to data collection forms can be requested from the corresponding author.

## Discussion

This field trial has a novel combination of aims: to evaluate simultaneously the efficacy of an innovative dengue vector control intervention and to develop methods and indices to anticipate changes in dengue transmission and predict impending outbreaks. Such objectives are in harmony with recent published recommendations on global frameworks on vector control and contingency planning for dengue outbreaks [39, 47] as well as recommendations from several review papers on these topics [16, 75]. If successful, results from this study will provide important information on dengue vector control and contribute to the further development of early warning systems and deployment of effective responses to dengue outbreaks.

Currently, the primary vector control methods used by the majority of public-funded dengue control programs are treatment of water storage containers with a larvicide (commonly temephos) and/or peridomestic space spraying of insecticides. Additionally, source reduction practices through community-based clean-up campaigns are common vector control interventions. Although these standard interventions are recommended by WHO [5], there is currently no clear evidence that they have any demonstrable effect on reducing dengue transmission [7, 9, 33].

A systematic literature review on the effectiveness of temephos found that as a single community-based intervention it controlled larvae for 2–3 months, depending on study design, local circumstances, water turnover rates, and season [7]. However, temephos appears not to work well in combination with other interventions, possibly due to an inordinate trust in (or reliance on) its effectiveness when used alone, poor implementation and coverage, and low acceptability for its use in drinking water [7]. The review concluded that many factors could influence the effectiveness of temephos, such as the degree of intervention coverage, quality of implementation and sustainability, how often treated water is exchanged, and characteristics and use of the target container itself.

A systematic review on the effectiveness of peridomestic space spraying (using pyrethroids, pyrethrins, or organophosphates) showed reductions in various entomological indices; however, the effect dissipated within a few days or weeks [9]. The authors concluded that the effectiveness of space spraying in reducing dengue transmission could not be confirmed and recommended more detailed research on its utility as a practical public health intervention. Container clean-up campaigns might be effective, although such interventions are often confounded by other simultaneous interventions, thus obscuring the effect of the source reduction campaign itself [75].

It appears that most current dengue vector control methods lack clear evidence of their effectiveness, which does not necessarily mean they are ineffective [75]. There have been few well-designed trials, and most have focused on measuring larval and pupal densities, which may not be epidemiologically reliable [16]. The current trial is therefore of great interest to the international dengue control community, as it will look at a much wider range of measures, including adult vector densities. Moreover, the novel use of a pyriproxyfen/spinosad combination in household water storage containers is a promising alternative to conventional vector control methods. Combining the two compounds in a large field trial under natural conditions has not yet been attempted. Furthermore, the two compounds complement each other in that one targets the mosquito larval stage (spinosad) and the other the pupal stage (pyriproxyfen); thus, they potentially provide long-term control in the environment and disease reduction [42, 44]. In Vietnam, pyriproxyfen used together with insecticide-treated covers of water storage containers successfully inhibited mosquito breeding for 5 months [76]. A small, simulated field trial using a pyriproxyfen/spinosad mixture reported that the mixture was effective for at least 8 months compared with 3 months for spinosad alone and 5 months for pyriproxyfen alone. In natural breeding sites the mixture remained effective for 4.5 months [42]. Both compounds are not toxic to humans or most non-target fauna [45, 46]. Pyriproxyfen also has an additional advantage in that it can be disseminated to other larval habitats by adult mosquitoes [30–32].

This trial is also designed to identify practical and sensitive entomological and immunological indicators for prediction of dengue transmission and increased risk for dengue outbreaks. The more accurate, timely, and site-specific the prediction, the greater the likelihood a control response would mitigate, if not prevent, the outbreak from occurring. The originality of this trial is that virological and immunological methods are used in combination with standard entomological measures in both intervention and control clusters. The Peru study mentioned previously [17] investigated the relationship between indicators of mosquito abundance and DENV infection. Although, it is probably one of the most comprehensive studies to date, such abundance-based indicators are not likely to be sensitive enough to detect changes in intensity of transmission. A better indicator would be to monitor adult mosquitoes for dengue viral infection, similarly as is done to assess malaria transmission risk using the entomological inoculation rate. RDTs can be used as a simple method to detect DENV antigen in mosquitoes [77, 78]. New methods to monitor DENV-infected adult *Aedes* densities using various trapping designs and RDTs have been proposed as a new paradigm in *Aedes* surveillance [79, 80].

Another potentially promising indicator is to measure the exposure of people to mosquito bites using human antibody response to mosquito salivary protein. A recent study carried out along the Thai-Myanmar border areas demonstrated that levels of IgG response were positively associated with anopheline vector abundance and the entomological inoculation rate [81]. The antibody response to *Ae. aegypti* whole saliva has been shown to be a quantitative biomarker of human exposure in Africa and South America [82, 83]. More recently, a salivary peptide (Nterm-34 kDa) was identified as a specific *Aedes* biomarker [84, 85]. The IgG immune response to Nterm-34 kDa salivary peptide is not expected to last for more than 15–30 days; hence, it represents a relevant temporal biomarker to assess recent relative exposure of humans to *Aedes* bites [84]. This peptide was used successfully as a short-time indicator to evaluate vector control interventions against *Aedes* exposure in Réunion Island [86].

In this trial, DENV detection in adults and pupae will be assessed in relation to the number of recent and subsequent confirmed dengue incidents in humans in the same locality. Human exposure to *Aedes* bites measured by IgG antibody response will be examined for correlation with dengue cases. Data will be analyzed to include socioeconomic factors and influence of environmental and seasonal fluctuations, such as rainfall, relative humidity, and ambient temperature. These parameters and specific measures have so far not been fully integrated in epidemiological risk assessments and epidemic forecasting.

The permanent staff of local public health departments, sub-district hospitals, and VHVs will collect data for all listed outcomes, thereby minimizing involvement of full-time trial project staff. This is intended, as much as possible, to allow national authorities to emulate the project procedures in follow-on surveillance and intervention activities or adoption of these methods into routine vector control program activities. The exception to this is molecular-based assay confirmation of DENV in human blood and mosquitoes and human antibody response to *Ae. aegypti* salivary peptides; this testing will be conducted by project staff.

### Study limitations

Several potential limiting factors may affect study outcomes. High spatial and temporal variation in dengue transmission dynamics may result in an insufficient number of incident infections to allow reliable associations between indices and dengue risk. This is why collections will be conducted over a 2-year period and in two urban areas to increase the potential of witnessing an upsurge in transmission as opposed to an interepidemic period. Nevertheless, the sample size required to detect significant differences in dengue incidence between the intervention and control arms was deemed unfeasibly large, thus relegating dengue incidence as a secondary outcome. Conversely, a dengue outbreak could likely overwhelm data collection systems used in the trial and further compel public health authorities to intervene with standard vector control interventions on a broad scale, thus potentially interfering with study outcomes. If outcome measures are substantially suppressed, this would negatively affect the power of the study.

Lastly, the proportion of asymptomatic (inapparent) and infectious persons in the study area may affect prediction outcomes, because they will not be captured by the data collection procedures used in the trial, although they may contribute to transmission [87]. Although the ratio of asymptomatic to symptomatic cases can be as high as 14:1 or higher, the epidemiological role of asymptomatic infections remains unclear [88].

### Trial status

At the time of submission of this manuscript, the trial has enrolled village clusters, requested household participation, and started baseline data collections in Khon Kaen (but not yet in Roi Et). Recruitment of patients has not started.

## Additional files

Additional file 1: SPIRIT checklist. (DOCX 53 kb)

Additional file 2: WHO Trial Registration Data Set (Version 1.3). (DOCX 21 kb)

Additional file 3: Consent forms. (DOCX 47 kb)

## Abbreviations

*Ae.*: *Aedes*
AI: Adult index
ARIMA: Autoregressive integrated moving average
ASTI: Adult sticky trap index
BI: Breteau index
CI: Container index
DENV: Dengue virus
DIR: Dengue incidence rate
DMC: Data Monitoring Committee
HH: Household
HI: House index
IAI: Infected adult index
IgG: Immunoglobulin G
IVM: Integrated vector management
kDa: kilodalton
KKUEC: Khon Kaen University Ethics Committee
MEI: Mosquito exposure index
NSD: Norwegian Centre for Research Data
OD: Optical density
ΔOD: Differential optical density
PBS: Phosphate-buffered saline
PPI: Pupae per person index
qPCR: Quantitative polymerase chain reaction
RDT: Rapid diagnostic test
RNA: Ribonucleic acid
RT-PCR: Reverse transcription polymerase chain reaction
SD: Standard deviation
TR: Immune threshold
USD: United States dollar
VHV: Village health volunteer
WHO: World Health Organization
