# Correction to: Assessing dengue transmission risk and a vector control intervention using entomological and immunological indices in Thailand: study protocol for a cluster-randomized controlled trial

**DOI:** 10.1186/s13063-018-3110-9

**Published:** 2018-12-24

**Authors:** Hans J. Overgaard, Chamsai Pientong, Kesorn Thaewnongiew, Michael J. Bangs, Tipaya Ekalaksananan, Sirinart Aromseree, Thipruethai Phanitchat, Supranee Phanthanawiboon, Benedicte Fustec, Vincent Corbel, Dominique Cerqueira, Neal Alexander

**Affiliations:** 1 Norwegian University of Life Sciences, Ås, Norway; 2 Khon Kaen University, Khon Kaen, Thailand; 3 HPV & EBV and Carcinogenesis Research Group, Khon Kaen University, Khon Kaen, Thailand; 4 Office of Disease Prevention and Control, Region 7, Khon Kaen, Thailand; 5 PT Freeport Indonesia/International SOS Indonesia, Kuala Kencana, Indonesia; 6 Kasetsart University, Bangkok, Thailand; 7 Université de Montpellier, Montpellier, France; 8 Institut de Recherche pour le Développement (IRD), Maladies Infectieuses et Vecteurs, Ecologie, Génétique, Evolution et Contrôle (MIVEGEC, UM1-CNRS 5290-IRD 224), Montpellier, France; 9 MRC Tropical Epidemiology Group, London, UK

**Correction to: Trials (2018) 19:122**

**https://doi.org/10.1186/s13063-018-2490-1**

In the original publication [1], the first of two objectives was to “Assess the effect of periodically treating water storage containers with a pyriproxyfen/spinosad combination on entomological and epidemiological outcomes”. However, spinosad will not be a part of the intervention. The approval by the Food and Drug Administration of the Ministry of Public Health, Thailand to use spinosad in this research project was delayed and not in place when the interventions were supposed to begin. Therefore, a decision was made to exclude spinosad in the trial and only use pyriproxyfen. The correct version of objective 1 should now read “Assess the effect of periodically treating water storage containers with pyriproxyfen on entomological and epidemiological outcomes”. The second objective remains the same: “Determine the most accurate and precise index or indices to predict variation in dengue incidence in time”.

The exclusion spinosad in the trial has implications for several sections in the original article. The word spinosad appears 18 times in the main text of document; in the abstract, background, methods, and discussion. The reader should be aware of this important change when reading the article and its implications for the trial and outcomes.
