# Supplementary material for: Assessing dengue transmission risk and a vector control intervention using entomological and immunological indices in Thailand: study protocol for a cluster-randomized controlled trial
Source: Trials. 2018 Feb 20;19:122. doi: 10.1186/s13063-018-2490-1 (PMC5819278; doi:10.1186/s13063-018-2490-1)
Supplement: Supplementary file 2 — WHO Trial Registration Data Set (Version 1.3). (DOCX 21 kb) [file 13063_2018_2490_MOESM2_ESM.docx]

**Additional file 2. WHO Trial Registration Data Set (Version 1.3) based on** [**http://www.who.int/ictrp/network/trds/en/**](http://www.who.int/ictrp/network/trds/en/)

| **#** | **Data category** | **Information** |
| --- | --- | --- |
| 1 | Primary Registry and Trial Identifying Number | ISRCTN Registry, ISRCTN73606171 |
| 2 | Date of Registration in Primary Registry | 23 June 2017 |
| 3 | Secondary Identifying Numbers | RCN250443, KKUEC HE601221, LSHTM Ethics 14275, REK 2017/1826 |
| 4 | Source(s) of Monetary or Material Support | Research Council of Norway |
| 5 | Primary Sponsor | Norwegian University of Life Sciences |
| 6 | Secondary Sponsor(s) | - |
| 7 | Contact for Public Queries | HJO [hans.overgaard@nmbu.no] |
| 8 | Contact for Scientific Queries | HJO [hans.overgaard@nmbu.no] |
| 9 | Public Title | - |
| 10 | Scientific Title | Assessing dengue transmission risk and a vector control intervention using entomological and immunological indices in Thailand: Study protocol for a cluster-randomized controlled trial |
| 11 | Countries of Recruitment | Thailand |
| 12 | Health Condition(s) or Problem(s) Studied | Dengue |
| 13 | Intervention(s) | Active comparator: Pyriproxyfen+spinosad in combination added to mosquito breeding containers every three months.  Control: Normal governmental dengue control activities |
| 14 | Key Inclusion and Exclusion Criteria | INCLUSION CRITERIA:  Village: Within ring roads of each strata (city), populated residential areas.  Cluster: All points of the cluster are at least 100 m from the nearest point of the village border.  Household: 1) Households that are permanently inhabited, 2) Households that are built or re-populated during the study period  Individuals: 1) In households where household head has signed informed consent for household to participate in project, 2) A travel history outside the village during the previous 7 days, 3) Chronic diseases such as HIV/AIDS or cancer, 4) Apparent inability to give informed consent, e.g., due to mental disability or other incapacity, or lack of a legally authorized representative, 5) Disease with similar symptoms to dengue but with evident alternative explanation, 6. Age <1 year old.  EXCLUSION CRITERIA:  Village: 1) Area <0.125 km2, 2) Number of houses<100, 3) Population <300, 4) Coverage of residential area 70-80% (scattered housing), 5) Non-residential areas, e.g. agricultural fields, airport, industrial areas, commercial areas, (e.g. shopping malls), government offices, lakes. army camps, hospitals and schools.  Household: 1) Apartment buildings, 2) Abandoned houses, 3) Non-permanent households.  Individual: 1) Refusal to participate in the study, 2) Age <1 year old. |
| 15 | Study Type | Interventional  Study design: Cluster randomised trial, no masking, single arm  Primary purpose: Prevention |
| 16 | Date of First Enrollment | July 2017 |
| 17 | Sample Size | 2 strata, 18 clusters per strata, 10 households per cluster, ca 4 persons per household: 2x18x10x4=1440. |
| 18 | Recruitment Status | Recruiting |
| 19 | Primary Outcome(s) | Adult Index (AI, the number of female adult Ae. aegypti and Ae. albopictus collected per house) |
| 20 | Key Secondary Outcomes | Dengue Incidence Rate (no. of confirmed dengue cases / observation-days of household populations).  Mosquito Exposure Index: a) Differential optical density for antibodies to Ae. aegypti saliva, and b) Proportion above the immune threshold for this assay.  Infected Adult Index (no. of DENV infected adult female Ae. aegypti and Ae. albopictus).  Adult Sticky Trap Index (total no. of Ae. aegypti and Ae. albopictus females collected by sticky traps per month). |
| 21 | Ethics Review | KKUEC HE601221 (approved 1 Sep 2017), LSHTM Ethics 14275 (approved 16 Aug 2017), REK 2017/1826 (in process) |
| 22 | Completion date | August 2019 (expected) |
| 23 | Summary Results | Not started yet |
| 24 | IPD sharing statement | Undecided |
