# Supplementary material for: Assessing dengue transmission risk and a vector control intervention using entomological and immunological indices in Thailand: study protocol for a cluster-randomized controlled trial
Source: Trials. 2018 Feb 20;19:122. doi: 10.1186/s13063-018-2490-1 (PMC5819278; doi:10.1186/s13063-018-2490-1)
Supplement: Supplementary file 3 — Consent forms. (DOCX 47 kb) [file 13063_2018_2490_MOESM3_ESM.docx]

Informed Consent Form

**Request for household participation in a research project**

**Project name**

***“Assessing dengue transmission risk and a vector control intervention using entomological and immunological indices in Thailand: A cluster-randomized controlled trial”***

| **Principal Investigator:**  Dr. Hans J. Overgaard, Norwegian University of Life Sciences, Norway | **Principal Thai Investigators (PI):**   - Assoc. Prof. Chamsai Pientong, Khon Kaen University, Khon Kaen, Thailand   - Dr. Kesorn Thaewnongiew, Office of Diseases Prevention and Control Region 7, Khon Kaen |
| --- | --- |

# This is a request for you to participate in a research study on dengue

# Criteria for participation

# Your village and house have been randomly selected to be part of the study.

# You have earlier been contacted by DENGUE-INDEX project staff and have preliminary agreed to participate in the study.

# Background and purpose

Dengue is a serious public health problem in Thailand. Dengue is caused by a virus transmitted by mosquitoes. This study will try to find better ways to control these mosquitoes. In this trial, we will test whether mosquito production can be reduced by applying two natural compounds in the water storage containers where mosquitoes breed. All containers in the houses that participate in this project will be treated by a combination of pyriproxyfen, which is an insect growth regulator, and spinosad, which is a natural bacterial product that stops mosquitoes from emerging. In addition, the study also intends to develop entomological and immunological indicators or indices that accurately represent dengue transmission. With such indices, it may be possible to develop early warning systems that can predict dengue epidemics. To achieve this purpose we want to recruit all subjects who live in the selected households and reported a fever. Some of them will have dengue and some not. We then collect other information on potential risk factors, such as patients’ age, travel history, previous dengue infection history, etc. As part of the study we will also test for other viruses, which are also transmitted by mosquitoes, such as chikungunya and Zika.

# What does the study entail for you?

Your household with all household members living here will participate in this study, which will last for two years.

**Household questionnaire.** You as the head of this household will be asked to answer a household questionnaire with questions on the number of people living there and socioeconomic status, including observations of type and quality of house structure and facilities.

**Disease surveillance**: If you or someone in your household will get a fever during the two-year study period, we would like that person to give a blood sample of 4 mL (4 cc) and answer a few simple questions. Village health volunteers will visit you each week to monitor any fever episodes. We will again ask for consent from each person who gets fever during the study. The best place to give blood is in the nearest sub-district hospital and we will help you to go there.

**Entomological survey**: Mosquito collections will be carried out in all participating households every four months. In addition, monthly collections will be done in three households per cluster, using the same households as the blood spot collections for logistical reasons. You will be informed if your household is among those where collections will be done every month.

**Exposure to mosquito bites:** To assess the level of exposure to dengue mosquito bites, blood spots on filter paper will be taken using a finger prick from each presumptive dengue case for immunological analysis. In addition, regular monthly blood spot collections will be done from two additional individuals (ideally one adult and one child 5-14 years old), always being the same each month, in each of three households per cluster. You will be informed if your household is among those where collections will be done every month. Selection of individuals will be based on their availability and willingness to participate; ideally, individuals who are at home most of the time**.**

**Potential advantages and disadvantages**

**Advantages:** You will have the result of a dengue Rapid Diagnostic Test to be sure if your fever is because of dengue. The results will be given to you just after the test. You will also be informed whether you have evidence of infection with other viruses transmitted by mosquitoes, in particular Zika and Chikungunya virus.

**Disadvantages:** You will be asked to provide blood spots on filter paper and 4 mL of blood. This requires the use of a needle, which may be slightly painful and there is a small probability of bruising, tingling or other unusual sensations, or fainting, especially if you are not used to giving blood. You may also be asked to give two blood spots on a paper by a finger prick. Interviewing you will take some of your time.

**Your responsibilities**

If you take part, we will expect you to: respect those involved in the study, be truthful when answering; and also let us know if you decide, at any time, not to continue in the study without stating any particular reason. You should also make sure that all your household members are aware and know that your household is part of the study.

If you, or anyone in your household, have a chronic disease (such HIV/AIDS) or another health condition that you think would preclude you to participate in the study, please let us know and you will not be part of the study. Any information you may have provided in relation to this is confidential and will not be revealed to anyone.

**Responsible project leaders and contact details**

If you later on wish to withdraw your consent or have questions concerning the study, please contact the Principal Thai Investigators (PIs) or their representatives:

- Prof. Chamsai Pientong, Department of Microbiology, Faculty of Medicine, Khon Kaen University, Khon Kaen, at this telephone number 081-262-3997.
- Dr. Kesorn Thaewnongiew of the Office of Diseases Prevention and Control Region 7, Khon Kaen, at this telephone number 095-492-5998.
- Office of The Khon Kaen University Ethics Committee in human research (Sub office) Room 5317, 3rd Floor Wadwichakarn Building , Faculty of Medicine, Khon Kaen University Tel. 67133 – 4 Mobile 089-7141913.

You will receive an information sheet, which you can bring back home and read it again at a later time.

**Consent for household participation in the study**

I am willing to participate in the study.

Date:

----------------------------------------------------------------------------------------------------------------

*(Signed by the project participant)*

Legal representative consent when this is warranted, either in addition to or in place of the participant’s consent.

Date:

----------------------------------------------------------------------------------------------------------------

*(Signed by representative, parents or guardian)*

I confirm that I have given information about the study.

Date:

----------------------------------------------------------------------------------------------------------------

(*Role in study of person giving information*)

Informed Consent Form

Request for individual participation in a research project

**Project name**

***“Assessing dengue transmission risk and a vector control intervention using entomological and immunological indices in Thailand: A cluster-randomized controlled trial”***

| **Principal Investigator:**  Dr. Hans J. Overgaard, Norwegian University of Life Sciences, Norway | **Principal Thai Investigators (PI):**   - Assoc. Prof. Chamsai Pientong, Khon Kaen University, Khon Kaen, Thailand   - Dr. Kesorn Thaewnongiew, Office of Diseases Prevention and Control Region 7, Khon Kaen |
| --- | --- |

This is a request for you to participate in a research study on dengue.

**Criteria for participation**

We are asking you to participate for three reasons:

1) You, or your child aged less than 18 years (if not your own child, the child you are accompanying), had a fever (which potentially could be dengue fever),

2) You are older than 1 years old,

# Background and purpose

Dengue is a serious public health problem in Thailand. Dengue is caused by a virus transmitted by mosquitoes. This study will try to find better ways to control these mosquitoes. In this trial, we will test whether mosquito production can be reduced by applying two natural compounds in the water storage containers where mosquitoes breed. All containers in the houses that participate in this project will be treated by a combination of pyriproxyfen, which is an insect growth regulator, and spinosad, which is a natural bacterial product that stops mosquitoes from emerging. In addition, the study also intends to develop entomological and immunological indicators or indices that accurately represent dengue transmission. With such indices, it may be possible to develop early warning systems that can predict dengue epidemics. To achieve this purpose we want to recruit all subjects who live in the selected households and reported a fever. Some of them will have dengue and some not. We then collect other information on potential risk factors, such as patients’ age, travel history, previous dengue infection history, etc. As part of the study we will also test for other viruses, which are also transmitted by mosquitoes, such as chikungunya and Zika.

# What does the study entail for you?

If you have a fever or have had a fever during the last week, we will ask you to go to the sub-district hospital to give a blood sample. We will take a 4 mL (4 cc) of your blood. From this blood sample we will:

1. Do a Rapid Diagnostic Test for dengue. You will receive the outcome of this test before you leave the hospital.
2. Do more dengue tests to see what, if any, type of dengue virus you have.
3. Test for exposure to mosquito bites.
4. Test for infection with other mosquito-transmitted viruses, especially Zika and Chikungunya. It may take some months to inform you of the outcome of this test.

If you choose not to participate you will receive standard care.

**Potential advantages and disadvantages**

**Advantages:** You will have the result of a dengue Rapid Diagnostic Test, which you may not have had otherwise. You will also be told whether you have evidence of infection with other viruses transmitted by mosquitoes, in particular Zika and Chikungunya virus.

**Disadvantages:** You will be asked to provide 4 mL of blood. This requires the use of a needle, which may be slightly painful and there is a small probability of bruising, tingling or other unusual sensations, or fainting, especially if you are not used to giving blood. Interviewing you will take some of your time.

**Your responsibilities**

If you take part, we will expect you to: respect those involved in the study, be truthful when answering; and also let us know if you decide, at any time, not to continue in the study without stating any particular reason.

**What will happen to the samples and the information about you?**

Your blood samples will be stored at a laboratory in Khon Kaen University, either in a freezer or on dried filter papers. Your personal information will be stored on the database, which can be accesses by the authorized persons only. The samples (blood) and data (personal and household information) that are registered about you will only be used in accordance with the purpose of the study as described above. The authorized person having access to biological materials is Assoc. Prof. Chamsai Pientong, Khon Kaen University. All the data and samples will be analysed without name, ID card number or other directly recognisable type of information. A code number links you to your data and samples by a list of names.

Only authorized project personnel will have access to the list of names and be able to identify you. This identifying information will be deleted by the end of year 2567 (2024 in Western calendar). If you agree to participate in the study, you also consent to your de-identified data being released to London School of Hygiene and Tropical Medicine (LSHTM) for further analysis. Your blood samples will not be released to other parties.

It will not be possible to identify you in the results of the study when these are published or stored in a data repository.

**Voluntary participation**

Participation in the study is voluntary. We don’t expect that the study will cause you any monetary expense and you will not receive any money for participating. You can withdraw your consent to participate in the study at any time and without stating any particular reason. This will not have any consequences for your further treatment. If you wish to participate, please sign this declaration of consent. If you agree to participate at this time, you may later on withdraw your consent without your treatment being affected in any way.

If you agree to participate in the study, you are entitled to have access to what information is registered about you. You are further entitled to correct any mistakes in the information we have registered. If you withdraw from the study, you are entitled to demand that the collected samples and data are deleted, unless the data have already been incorporated in analyses or used in scientific publications.

If you have a chronic disease (such HIV/AIDS) or another health condition that you think would preclude you to participate in the study, please let us know and you will not be part of the study. Any information you may have provided in relation to this is confidential and will not be revealed to anyone.

**Responsible project leaders and contact details**

If you later on wish to withdraw your consent or have questions concerning the study, please contact the Principal Thai Investigators (PIs) or their representatives:

- Assoc. Prof. Chamsai Pientong, Department of Microbiology, Faculty of Medicine, Khon Kaen University, Khon Kaen, at this telephone number 081-262-3997.
- Dr. Kesorn Thaewnongiew of the Office of Diseases Prevention and Control Region 7, Khon Kaen, at this telephone number 095-492-5998.
- Office of The Khon Kaen University Ethics Committee in human research (Sub office) Room 5317, 3rd Floor Wadwichakarn Building , Faculty of Medicine, Khon Kaen University Tel. 67133 – 4 Mobile 089-7141913.

You will receive an information sheet, which you can bring back home and read it again at a later time.

**Consent for participation in the study**

I am willing to participate in the study.

Date:

----------------------------------------------------------------------------------------------------------------

*(Signed by the project participant)*

Legal representative consent when this is warranted, either in addition to or in place of the participant’s consent.

Date:

----------------------------------------------------------------------------------------------------------------

*(Signed by representative, parents or guardian)*

I confirm that I have given information about the study.

Date:

----------------------------------------------------------------------------------------------------------------

----------------------------------------------------------------------------------------------------------------

(*Role in study of person giving information*)

Informed Assent Form

Request for individual participation in a research project

Assent: for those aged 7-13 years

**Project name**

***“Assessing dengue transmission risk and a vector control intervention using entomological and immunological indices in Thailand: A cluster-randomized controlled trial”***

| **Principal Investigator:**  Dr. Hans J. Overgaard, Norwegian University of Life Sciences, Norway | **Principal Thai Investigators (PI):**   - Assoc. Prof. Chamsai Pientong, Khon Kaen University, Khon Kaen, Thailand - Dr. Kesorn Thaewnongiew, Office of Diseases Prevention and Control Region 7, Khon Kaen |
| --- | --- |

I understand that this research project is trying to find better ways to reduce risk of dengue by controlling mosquitoes.

I understand that I will be asked some questions and requested to give 4 mL (4 cc) of blood, about a teaspoon, via a needle.

I know that I can choose whether or not to take part in this research project. I know that I can stop taking part whenever I want. I have read this sheet and understand it. Any questions I have now have been answered and I know that I can ask whatever questions I may have later.

**I agree to take part in this research study YES NO**

Signature of the person aged 7-13 years

Date:

----------------------------------------------------------------------------------------------------------------

*(Signature)*

----------------------------------------------------------------------------------------------------------------

*(Full name in legible letters)*

I confirm that I have given information about the study.

Date:

----------------------------------------------------------------------------------------------------------------

----------------------------------------------------------------------------------------------------------------

Role in study of person giving information

Informed Consent Form

Request for blood spot collections

Project name

***“Assessing dengue transmission risk and a vector control intervention using entomological and immunological indices in Thailand: A cluster-randomized controlled trial” - DENGUE INDEX Study 2***

| **Principal Investigator:**  Dr. Hans J. Overgaard, Norwegian University of Life Sciences, Norway | **Principal Thai Investigators (PI):**   - Assoc. Prof. Dr. Chamsai Pientong, Khon Kaen University, Khon Kaen, Thailand - Dr. Kesorn Thaewnongiew, Office of Diseases Prevention and Control Region 7, Khon Kaen |
| --- | --- |

This is a request for you to participate in a research study on dengue.

**Criteria for participation**

We are asking you to participate for three reasons:

1. Your household is located in the selected cluster of the DENGUE INDEX Study 2 and the head of your household agrees to participate in the study,
2. You are 5 year old or older,
3. You spend most of time in your house.

# Background and purpose

The study intends to develop an immunological indicator that accurately represents the exposure to dengue vector bites and may reflect dengue transmission. The immunological indicator will also be used to evaluate a vector control intervention. To achieve this, we want to take a blood sample from 2 people living in this household. The two household members selected for blood spot sampling will be an adult and a child not younger than 5 years old. Participating in this is voluntary, but you will be compensated with 50 bath each time you provide dry blood spots. The blood collected will be used to see if there are differences between households with dengue and those without dengue and to evaluate the vector control intervention.

# What does the study entail?

If you agree to take part in the study, we would take 2 droplets of your blood by a fingertip prick every month for the total duration of the study (2 years). We will also ask you a few questions.

**Potential disadvantages**

To provide blood we need to puncture the skin on your fingertip with a needle, which may be slightly painful. There is a small chance of bruising, tingling or other unusual sensations, and fainting, especially if you are not used to providing blood. The questions will take a little bit of your time.

**Your responsibilities**

If you take part, we will expect you to respect those involved in the study; tell us if you don’t want to give any of the information requested, but otherwise be truthful when answering; and also let us know if you decide, at any time, not to continue in the study.

**What will happen to the samples and the information about you?**

Your blood samples will be stored at a laboratory in Khon Kaen University on dried filter papers. Your personal information will be stored on paper in a locked safe and on a locked computer at Khon Kaen University. The samples (blood) and data (personal and household information) that are registered about you will only be used in accordance with the purpose of the study as described above. All the data and samples will be analysed without name, ID card number or other directly recognisable type of information. A code number links you to your data and samples by a list of names. The information will be given in the Household Blood Spot Questionnaire that you will be asked to fill out after signing this consent form.

It will not be possible to identify you in the results of the study when these are published.

**Voluntary participation**Participation in the study is voluntary. You will have no monetary expenses and you will not receive any money for participating. You can withdraw your consent to participate in the study at any time and without stating any particular reason. If you wish to participate, please sign this declaration of consent. If you agree to participate at this time, you may withdraw your consent later without being affected in any way.

If you agree to participate in the study, you are entitled to have access to the information registered about you. You are further entitled to correct any mistakes in the information we have registered. If you withdraw from the study, you are entitled to demand that the collected samples and data are deleted, unless the data have already been incorporated in analyses or used in scientific publications.

**Responsible project leaders and contact details**

If you later on wish to withdraw your consent or have questions concerning the study, please contact the Principal Thai Investigators (PIs) or their representatives:

- Assoc. Prof. Dr. Chamsai Pientong, Department of Microbiology, Faculty of Medicine, Khon Kaen University, Khon Kaen, at this telephone number 081-2623997.
- Dr. Kesorn Thaewnongiew of the Office of Diseases Prevention and Control Region 7, Khon Kaen, at this telephone number 061-0311469.
- Office of the Khon Kaen University Ethics Committee in human research (Sub office) Room 5317, 3rd Floor Wadwichakarn Building , Faculty of Medicine, Khon Kaen University Tel. 67133 – 4 Mobile 089-7141913.

**Consent for participation in the study**

I am willing to participate in the study.

Date:

----------------------------------------------------------------------------------------------------------------

*(Signed by the project participant)*

Proxy consent when this is warranted, either in addition to or in place of the participant’s consent.

Date:

----------------------------------------------------------------------------------------------------------------

*(Signed by representative)*

I confirm that I have given information about the study.

Date:

----------------------------------------------------------------------------------------------------------------

----------------------------------------------------------------------------------------------------------------

(*Role in study of person giving information*)
